# Treatment of Herpes Zoster

**Published:** 1882-01

**Authors:** John Boardman


### Treatment of Herpes Zoster.

Reported by John Boardman, M. D.

*Case*—M. D. came into my office with an interrupted band of herpes zoster, extending on the right side the vertebræ to near the pubis, which he discovered two days previous. I ordered him to use the following prescription:

℞ Carbolic acid ʒii.

Ol. oliv ℥i.

Signa: Rub well on the parts two or three times daily.

I did not see him for a week when the eruption had disappeared, only a few dry crusts remaining. He stated that after the second application the burning of the parts was relieved.

My purpose in this brief report is to direct the attention of the profession to the use of carbolic acid in the treatment of "shingles," as it has been very successful in my hands.

